# Ventricular Tachycardia Storm Presenting as Vague Complaints to the Emergency Department

**DOI:** 10.5811/cpcem.2019.5.43052

**Published:** 2019-07-08

**Authors:** Ravneet Kamboj, Andy C. Bunch, Robert C. Bernstein, Francis L. Counselman

**Affiliations:** * Eastern Virginia Medical School, Department of Emergency Medicine, Norfolk, Virginia; † Sentara Cardiology Specialists, Department of Cardiology, Norfolk, Virginia; ‡ Emergency Physicians of Tidewater, Norfolk, Virginia

## Abstract

We present the case of a 75-year-old man with vague symptoms and hypotension found to be in electrical storm secondary to sustained ventricular tachycardia. The patient did not respond to intravenous amiodarone, magnesium, lidocaine, or four cardioversion attempts. This case illustrates the challenges in managing patients with electrical storm presenting to the emergency department.

## INTRODUCTION

We present the case of a 75-year-old man who presented to the emergency department (ED) with the primary complaints of lightheadedness and blurred vision. The patient was found to be hypotensive and in ventricular tachycardia (VT) storm. Despite optimal medical therapy with intravenous amiodarone, magnesium, lidocaine, and a total of four cardioversions, the patient remained in sustained VT or electrical storm. This is defined as three or more sustained episodes of VT, ventricular fibrillation (VF), or appropriate implantable cardioverter-defibrillator (ICD) shocks within a 24-hour period.^1^,^2^,^3^ VT, as in this case, is usually the abnormal rhythm, but VF can occur as well. Emergency physicians need to consider electrical storm in the setting of sustained VT, VF, or appropriate ICD shocks.

## CASE REPORT

A 75-year-old man presented to the ED complaining of lightheadedness and blurred vision. The patient had a history of myocardial infarction, deep venous thrombosis, and hyperlipidemia. He stated he had taken a sildenafil pill earlier in the day. A few minutes after taking it, he became dizzy, lightheaded and experienced blurred vision. The patient denied chest pain, shortness of breath, palpitations, or nausea and vomiting. He denied extremity numbness, weakness, or change in speech. The patient’s medications included warfarin 2.5 milligrams (mg) daily, simvastatin 20 mg daily, and sildenafil 100 mg as needed.

Physical exam revealed a pulse of 141 beats per minute, respiratory rate of 18 breaths per minute, blood pressure 84/48 millimeters of mercury (mmHg), 96% oxygen saturation on room air, and that he was afebrile. The cardiac monitor revealed a wide complex tachycardia consistent with VT. He appeared comfortable and was able to converse without any problem. The head, eyes, ears, nose, and throat exam was normal. Examination of the heart revealed a tachycardic, regular rhythm without murmurs, rubs, or gallop. Auscultation of the lungs revealed clear, bilateral breath sounds. The abdomen was soft, nontender, and without guarding or rebound. The neurologic exam was completely normal.

A stat electrocardiogram (ECG) was obtained, blood drawn, an intravenous (IV) line established, and a portable chest radiograph (CXR) ordered. The ECG showed VT (Image). The patient was given amiodarone 150 mg IV and two grams of magnesium IV, without any change in the rhythm or blood pressure. Given his hemodynamic instability, the patient was given midazolam one mg IV in preparation for synchronized biphasic cardioversion. The pads were placed in the anterolateral position, and the patient was cardioverted with 100 joules (J), followed by 200 J, without any change in condition. The patient was cardioverted again at 360 J, without any change in rhythm or blood pressure. Finally, the pads were changed to an anterior/posterior placement and the patient cardioverted at 360 J, again without change. A one-liter bolus of Ringers lactate solution IV was administered.

At this point, cardiology was consulted; they recommended giving amiodarone 300 mg IV (in addition to the initial 150 mg given). This was administered without any change in the patient’s condition. The cardiologist then evaluated the patient in the ED and gave adenosine six mg IV, followed by 12 mg IV, without effect. Cardiology then ordered lidocaine 100 mg IV bolus, again without any change in the patient’s status.

The basic metabolic profile and troponin I and T were normal. The patient was adequately anticoagulated with an international normalized ratio of 3.5 seconds. The complete blood count, hepatic panel, and magnesium level were all normal. The CXR showed streaky opacities in the right midlung zone, representing atelectasis, along with emphysematous changes.

The patient was admitted to the cardiac care unit with a diagnosis of VT refractory to amiodarone and cardioversion. He was continued on an amiodarone, lidocaine, and heparin IV drip. He was also started on a phenylephrine IV drip to keep the mean arterial pressure above 65 mmHg. Review of his most recent echocardiogram (ie, six months prior) showed a large area of thinning and akinesis involving the distal septum, apical and inferoapical walls, with an ejection fraction of 40%. Serial cardiac troponins were elevated, but were thought to be secondary to the multiple cardioversions in the ED.

The patient remained in VT, requiring IV pressor support for hypotension, but was otherwise asymptomatic. An electrophysiologist was consulted and recommended VT ablation with Impella support. The next morning, the patient was taken to the electrophysiology suite and intubated. Synchronized biphasic cardioversion at 125 J was performed, resulting in asystole. Atrial and ventricular pacing was immediately performed and capture obtained; the Impella device was placed and proper positioning confirmed. The patient developed VT, which was terminated with a single biphasic cardioversion. The decision was made to abandon the ablation procedure due to the development of severe biventricular failure with low cardiac output. The patient was started on IV dobutamine and norepinephrine for the low cardiac output and weaned off the phenylephrine.

**CPC-EM Capsule**

What do we already know about this clinical entity?

Electrical storm is defined as three or more sustained episodes of ventricular tachycardia (VT), ventricular fibrillation (VF), or appropriate implantable cardioverter - defibrillator shocks within a 24-hour period.

What makes this presentation of disease reportable?

Despite the fact this patient was hypotensive and in sustained ventricular tachycardia, he only complained of lightheadedness and blurred vision.

What is the major learning point?

Electrical storm can present with only vague symptoms and be resistant to traditional medical and electrical therapy, and must be considered in the differential diagnosis of sustained VT or VF.

How might this improve emergency medicine practice?

Emergency physicians must be aware of this uncommon, but life-threatening presentation of VT or VF, and how to manage appropriately.

Cardiothoracic surgery was consulted for possible surgical options. After careful review, given his condition and past medical history, they felt the patient was not an appropriate candidate for a durable mechanical assist device (i.e., ventricular assist device). Similarly, they did not think he was a candidate for extracorporeal membrane oxygenation, as he would have no exit strategy. The family made the decision to provide comfort care only. The Impella device was turned off and the patient extubated; he died shortly afterwards.

## DISCUSSION

This patient suffered from the electrophysiological phenomenon known as electrical storm. It is defined as three or more sustained episodes of VT, VF, or appropriate ICD shocks within a 24-hour period.^1^,^2^,^3^ Ventricular tachycardia is the culprit arrhythmia in the majority of cases, but VF can also be seen. In one study examining the causative arrhythmia in patients with electrical storm by interrogation of their ICD, VT was identified as the causative arrhythmia in 52% of cases, and VF in the remaining 48%.^4^ Interestingly, previous smaller studies had identified VF as the cause in only 14–40% of cases.^5^,^6^,^7^ Sustained VT presenting as electrical storm is usually monomorphic and associated with structural heart disease. It is typically due to electrical wave front re-entry around a fixed anatomic barrier, such as scar tissue following a myocardial infarction.^3^ In this situation, the abnormal re-entrant circuit is initiated and maintained due to the abnormal conduction present in scarred myocardium.^8^ The severity of the presentation and degree of hemodynamic compromise depends on several factors, including the ventricular rate, left ventricular function, the presence and degree of heart failure, and any loss of atrioventricular synchrony.^3^,^9^

In contrast, polymorphic VT is most often associated with acute ischemia, but can occur in its absence. Other risk factors for polymorphic VT include a prolonged QT interval, myocarditis and hypertrophic cardiomyopathy. If the electrical storm is secondary to polymorphic VT with a long QT interval, acquired causes must be considered, including electrolyte imbalance (i.e., hypokalemia, hypomagnesemia), hypothyroidism, and medications that prolong the QT interval (i.e., erythromycin).^3^ Similar to polymorphic VT storm, VF storm can occur, with ischemia as the primary cause. In general, in the setting of an acute coronary syndrome, the electrical storm most likely involves polymorphic VT or VF, as opposed to monomorphic VT. Additional risk factors for polymorphic VT/VF storm include history of hypertension, prior myocardial infarction, ST-segment changes at presentation, and chronic obstructive pulmonary disease. 1

One of the diagnostic challenges of electrical storm is its ability to present with a wide variety and severity of symptoms, ranging from lightheadedness and palpitations, to chest pain and cardiac arrest. In our case, the patient presented with vague, nonspecific complaints. Our patient specifically denied chest pain, shortness of breath, or palpitations. He also did not appear to be in any acute distress and was oriented, calm, and conversant throughout his ED visit, despite being tachycardic and hypotensive.

The first step in successfully managing these patients is correctly diagnosing the causative arrhythmia. In addition to the three ventricular arrhythmias previously discussed, the emergency physician must be able to distinguish supraventricular tachycardia (SVT) with aberrancy from VT. This most often occurs in patients with a bundle branch block, a ventricular preexcitation syndrome (i.e., Wolff-Parkinson-White), or a rate-related aberrancy.^3^ It is critical the clinician not use the patient’s hemodynamic status to distinguish between the two. Patients with VT storm can present hemodynamically stable and with only vague, nonspecific complaints. It is much safer to assume that a patient presenting with an ambiguous, wide-complex tachycardia has VT, especially if they have a history of structural heart disease.^3^ Treating SVT with aberrancy with a calcium-channel blocker in a patient actually experiencing VT can result in cardiac arrest and death.

Next, the clinician needs to determine if the patient is hemodynamically stable. For hemodynamically unstable patients with VT or VF, cardioversion or defibrillation is the initial treatment. If the patient is awake and mentating, consider administering a short-acting benzodiazepine (i.e., midazolam) prior to cardioversion. One case report found the use of propofol for sedation prior to cardioversion was associated with both the conversion and suppression of VT in a patient with electrical storm.^8^ The initial shock should be with 200 J on a biphasic defibrillator, or 360 J for a monophasic. The usual advanced cardiac life support (ACLS) algorithm is followed, including high-quality cardiopulmonary resuscitation, airway management, epinephrine, and amiodarone. If the patient is hemodynamically stable, it is important to distinguish monomorphic VT from polymorphic VT. While the initial treatment for the two can be the same (i.e., IV amiodarone), if polymorphic VT is the causative rhythm, then the provider needs to simultaneously consider (and treat if present) reversible causes, such as electrolyte abnormality or ischemia.

Given the infrequency of electrical storm, early cardiology consultation is recommended. Increased sympathetic activation has been implicated in the generation of electrical storm, whereas sympathetic blockade has been shown to prevent VF and sudden cardiac death.^8^ One study comparing sympathetic blockade for the treatment of VT storm to standard ACLS therapy found that sympathetic blockade with left stellate ganglion blockade, esmolol or propranolol significantly reduced both the number of VT/VF episodes and the mortality rate.^8^,^10^

After initial stabilization, patients should undergo catheter ablation of the arrhythmogenic foci of their electrical storm. They should be continued on antiarrhythmic medication, given the risk of recurrence of ventricular tachydysrhythmias. Unfortunately, patients who survive electrical storm are at much higher risk of recurrent ventricular tachydysrhythmias and death.

## CONCLUSION

Electrical storm is an electrophysiologic condition consisting of three or more sustained episodes of VT, VF or appropriate ICD shocks within a 24-hour period.^1^,^2^ It can present with a wide variety of clinical complaints, from mild to life-threatening, and these patients are at high risk for acute decompensation and death. Emergency physicians must be able to recognize and appropriately treat electrical storm. Initial therapy is guided by the patient’s presenting arrhythmia, hemodynamic status, standard ACLS therapy, and early cardiology involvement.

## Figures and Tables

**Image:**
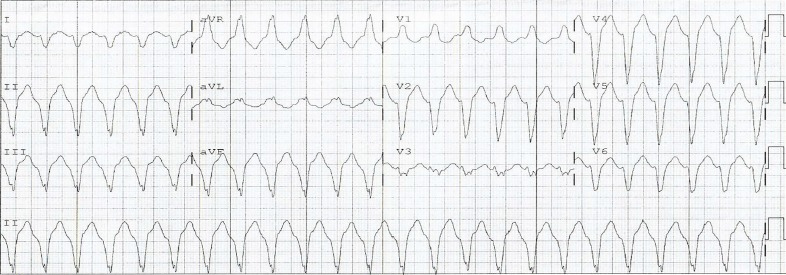
Electrocardiogram demonstrating monomorphic ventricular tachycardia in a patient with electrical storm.
